# Priorities challenges and opportunities of physical therapists who work with people that experience persistent pain

**DOI:** 10.1016/j.bjpt.2026.101607

**Published:** 2026-06-19

**Authors:** David S Kennedy, Tania Gardner, Sophie Shephard, Ian W Skinner

**Affiliations:** a Neurological and Pain Rehabilitation Laboratory, School of Exercise Science, Physical and Health Education. University of Victoria, Victoria, BC, Canada; b Sydney School of Health Sciences, Faculty of Medicine and Health, The University of Sydney, Sydney, NSW, Australia; c School of Allied Health Exercise and Sports Science, Charles Sturt University, Wagga; d School of Allied Health Exercise and Sports Sciences, Charles Sturt University, Port Macquarie, NSW, Australia

**Keywords:** Chronic pain, Clinical competence, Complex systems, Health care delivery, Physical therapy

## Abstract

**Background:**

Persistent pain is a complex condition that places substantial demands on physical therapists and healthcare systems. Understanding how clinicians experience and navigate these complexities is essential for improving care.

**Objective:**

To investigate the challenges, priorities, and opportunities from the perspective physical therapists who work with people experiencing persistent pain.

**Methods:**

We conducted focus groups with physical therapists working in pain management settings. Participants responded to four open-ended discussion questions in written and verbal formats. Data were analyzed using systematic text condensation to identify themes.

**Results:**

Sixty-six physical therapists across eight focus groups participated. An overarching theme of "complexity of pain" emerged, reflecting its influence across all aspects of care. Eleven interrelated subthemes were identified: clinical skills and education; funding models; professional networking and referral pathways; health professional awareness of evidence-based pain management; community awareness of evidence-based pain management; access to care; physical therapy advocacy and leadership; patient engagement and expectations; clinical time; clinician and patient emotional wellbeing; and clinical and social complexity.

**Conclusion:**

From the perspectives of Australian physical therapists, the complexities of managing persistent pain are deeply interrelated and extend across the healthcare system. These challenges are not attributable to pain complexity alone but reflect systemic issues spanning education, funding, access, and advocacy. Addressing these challenges requires coordinated action at individual, organizational, and societal levels.

## Highlights

• Persistent pain demands a systems approach spanning individual to societal levels.
• Education and mentorship are needed to support PTs in psychologically informed care.
• Better funding models are needed for adequate time and resources in pain management.
• The profession must drive systemic change in training, resources, and funding.

## Introduction

A major health burden, chronic pain is a debilitating and complex human experience that reduces the health and wellbeing of individuals and has considerable economic and social implications through direct health care costs and lost productivity.^1^, ^2^, ^3^, ^4^, ^5^ In Australia, the cost of chronic pain is estimated at $80 billion (2018) - more than heart disease, cancer, and diabetes combined.^6^ Evidence supports the interdisciplinary management of persistent pain, recommending an active approach that acknowledges and addresses the complexity and multifactorial nature of pain.^7^^,^^8^

Previous research has identified barriers to effective interdisciplinary pain care at the system, provider, and patient levels: for example, inconsistent or fragmented care, limited or poor access to community services and resources, opioid deprescribing mandates, a lack of health professional pain education, difficulties with interprofessional communication, the cost of treatment, and widespread patient and provider misconceptions about pain.^9^^,^^10^ Nurse practitioners often report that patients have either a lack of access to or an unwillingness to engage in nonpharmacologic management.^11^ Patients have also highlighted issues with long wait times for specialist appointments, short consultation times, and difficulties achieving an integrated interdisciplinary approach with consistent communication between professionals.^12^ Furthermore, these challenges are intensified in rural and regional Australia, where resources are limited and coordinated access to multidisciplinary services is particularly difficult.^13^^,^^14^ While some barriers are common across healthcare settings, different professions and clinical contexts face unique challenges.

Physical therapists working with people experiencing persistent low back pain often feel they lack the necessary knowledge and skills to address the cognitive, social, and psychological factors that influence pain, sometimes viewing these factors as beyond their scope of practice.^15^ Furthermore, early-career physical therapists, in particular, describe gaps in pain education and confidence in psychologically informed practice.^16^ Indeed, engaging patients in active pain self-management strategies and challenging their unhelpful beliefs about pain can be difficult, especially when patients expect physical therapists to "fix" their pain.^16^^,^^17^ Moreover, pain-related distress and emotional responses add to the complexity of care for physical therapists, who often feel frustrated by perceived gaps in their knowledge, training, and skills in addressing these psychological aspects of pain management.^18^ Many practitioners may feel wary or avoid psychologically informed practices altogether.^19^^,^^20^

While these studies illustrate some of the challenges, a more intentional and comprehensive exploration of physical therapists’s experiences is needed to document the unique barriers faced when working with people living with persistent pain, to highlight gaps in current training and support systems, and guide the development of tailored interventions to strengthen physical therapists' capacity to address the multifaceted needs of this population. Thus, this study aimed to explore the experiences of Australian physical therapists working with people experiencing persistent pain. The specific objectives were to investigate the challenges, priorities, and opportunities from the perspective of Australian physical therapists who work with people experiencing persistent pain.

## Methods

This was a pragmatic, exploratory, qualitative study using small group discussions analyzed via systematic text condensation to identify common themes across physical therapists’ perspectives on working with people experiencing persistent.

The study was informed by a pragmatic epistemology and a constructivist ontology, recognizing that knowledge about clinical practice is co-constructed through interaction and grounded in practitioners’ experiential understanding. Systematic Text Condensation (STC) aligns with this approach as a descriptive, phenomenologically inspired analytic framework emphasizing participants’ expressed meanings over theoretical interpretation.

The study follows the Standards for Reporting Qualitative Research (SRQR).^21^ Ethical approval was obtained from the University of Technology Human Research Ethics Panel.

### Participants

Participants were included if they fulfilled the following criteria: currently registered physical therapist in Australia or completing training to become a registered physical therapist in Australia, currently working or have worked in the past 12 months with patients who experience persistent pain.

### Recruitment

The sample of participants was one of convenience from a Physical therapy Pain Day Forum held in Sydney, Australia in December 2019. The Physical therapy Pain Day was organized by local physical therapists with support from the Australian Physical therapy Association. The aim of the day was to provide a forum for discussion on matters specifically related to pain management for physical therapists. There was a variety of guest speakers and presentations. There was no fee to attend the day. Permission was sought from the organizing committee to conduct the data collection as part of the day.

At the beginning of the day, a member of the organizing committee introduced the study to attendees who were given a participant information sheet. It was emphasized that participation was entirely voluntary. Attendees were given the opportunity to ask questions either privately during morning tea or in a public forum after the morning tea break. Those wanting to participate then attended the designated study discussion session. Consent forms were signed, and participants took part in the small group discussions.

### Question development

To facilitate the participant discussion addressing the research question, the research team developed four open-ended stimulus questions (Table 1) grounded in the study's objectives. The development process involved input from experienced physical therapists and the research team to ensure the questions were comprehensive and aligned with the study's aims. The final questions were designed to elicit detailed and specific responses, encouraging participants to share their perspectives and experiences in a structured manner.

**Table 1:** Stimulus questions.

1	What are the challenges that physical therapists face in delivering care for people with chronic pain? Prompt: How can these challenges be overcome? Be as specific as possible.
2	What are the opportunities for physical therapists for improving the delivery of care for people with chronic pain? Prompt: What needs to happen to ensure these opportunities are met? Be as specific as possible.
3	What are the priorities that need to be addressed to enhance the service delivery of people with chronic pain? Prompt: What needs to happen to address these priorities? Be as specific as possible.
4	What other issues and concerns do physical therapists have in their day to day management of people with chronic pain? Prompt: This is your opportunity to address any broader issues that have not previously come up that you feel need to be documented and raised in the literature.

### Data collection: small group discussions

The research team asked the participants to organize themselves into small groups of up to 10 people. Participants were encouraged to join with people that worked in different settings, or who they did not know. To describe the sample, participants completed a brief demographic questionnaire (Appendix A).

Each participant was given a copy of the four questions (Table 1). A scribe was self‑nominated for each group. There were four questions with ten minutes allocated to each question. Each question also contained an additional prompt to focus the conversations. Each group independently discussed each question with the scribe taking notes as the discussion took place. After eight minutes groups were asked to ensure they had provided a response to the specific question. Specific information provided to the group members and the scribes is available in Appendix B There was no audio or video recording of the small group discussion. Audio recording was not undertaken to preserve the informal, collegial nature of the forum and support participant comfort in an open professional setting. Each group used a standardized note-taking template (Appendix B) to capture key discussion points and illustrative quotes. Immediately following the session, notes were cross-checked with participants by the research team to ensure accuracy and consistency.

The researchers were physical therapists and educators with experience in pain management and professional training. No researcher had a prior clinical relationship with participants. Reflexive attention was given to how these professional backgrounds could influence interpretation. Team discussions were used to mitigate potential bias in data generation and analysis.

### Data analysis

To address the aims and objectives the data was analyzed using systematic text condensation (STC) for qualitative data.^22^ Systematic text condensation is appropriate for cross section data which involves inductive coding across different cases and does not quantify data. Questions response forms completed by the scribes were transcribed into word documents and checked by a second researcher.

For the first step and in accordance with systematic text condensation, initially three members of the research team (DK, TG, IS) reviewed and discussed the data to gain first impressions and to make sense of the data (Total Impression). Data from the word documents was then transposed to an excel workbook. The text was separated into individual cells based on discrete, fragmented ideas, relevant to the research questions.

For the second step, two reviewers (DK, TG) independently coded each discrete unit to identify meaning within the data within a decontextualized framework (Condensation). These codes were then reviewed by the research team (DK, TG, IS) with iterative coding and review taking place (Identifying and Sorting Meaning Units).

For the third step we established broad themes with specific subgroups based on the decontextualized codes. These were reviewed by the full research team (DK, TG, SS, IS).

In the fourth step the themes and data were reconceptualized within the context of the research and as they relate to the research questions (Synthesizing). This required iterative review and discussion of the data, themes, and context in relation to the research questions.

We were able to extract the most relevant content and meaning from the data and present cross-case thematic summaries. The final themes and summaries were reviewed and agreed by the research team.

## Results

All 66 participants who signed a consent form completed the data collection. There were eight groups (range 6–9 participants per group). Participants identified as being either Female (n = 48, 73%) or Male (n = 18, 27%) with no other responses reported. The mean years of employment as a physical therapist was 14.3 (SD = 11.4). For 23 (35%) participants >10% of their patient load involved a Workcover claim. The primary place of employment of participants is available in Table 2. Participants represented a range of experience (i.e., 1–42 years, median of 12), clinical settings, including metropolitan (n = 61, regional (n = 3), and rural areas (n = 2), with the majority working in the public hospital setting (see Table 2 for details).

**Table 2:** Primary place of employment for participants.

*Primary place of employment*	N = 66	Percentage
Public Hospital (Non pain unit)- including Emergency, outpatient.	31	47%
Tertiary Public Pain Clinic	7	11%
General private practice	7	11%
Community inc aged care	7	11%
Secondary Public Pain Clinic	6	9%
Private Pain Clinic	3	5%
Inpatient Rehabilitation	3	5%
Other	2	3%

The *complexity of pain* was the overarching theme that arose from the analysis. Within that, there were eleven themes that addressed the research aim and objectives. Table 3 outlines the eleven themes, key quotes as evidence of the theme, and the coverage (i.e., the percent of responses from all text data analyzed) of the theme across the data. While systematic text condensation does not specifically call for coverage of the themes, we felt it was useful to the reader to present the information as it reflects the consistency of the theme across discussions that took place within the groups. Details of the themes, solutions, or suggestions from participant responses via systematic text condensation follows.

**Table 3:** Themes, key quotes as evidence of the theme, and the coverage across responses.

	Theme	Coverage	Evidence (verbatim snippets from written responses)
1	Clinical Skills and Education	17%	- Undergrad curriculum to include pain + communication skills - Continuing local education to be prioritized - Lack of skill set within the profession, not really addressed in undergrad. The students asked us and we don’t understand either! - Education: physio level of knowledge about chronic pain - Education – physio/ GP- Community- all society - Access to current research + information - Pain website, research, all in one place, physio specific - Debriefing with colleagues - Lack of knowledge + scope of practice limitations - Expectations of providing excellent specialist treatment as a junior clinician - Medication training/ education for physical therapists
2	Funding Models	15%	- Health fund acknowledgement that some patients need more/ different care that could be cost effective in the long term - Aged care funding for chronic pain management- only for heat packs + massage, nil funding for more active and functional treatments - Look at other models of care around the world that are holistic. Work for complex pain conditions - Financial viability of private practitioners to treat chronic pain patients - Health funds: Recognize chronic pain/ understanding complexities; APA; Government; Cost of chronic pain/ investment - Funding, increased awareness of billing structures for increased consultation times. Streamlining non-clinical tasks to maximize time with patients. - Increase utilization of group therapies.
3	Professional Networking and Referral Pathways	13%	- Specialist services allow for appropriate triaging of patients + address the issues in a more timely manner - Orthopedic screening parallel clinics. Opportunity! Given physio in general is way ahead of managing these patients and increasing population of this patient type, funding models must change - Communication between public + private - Have a database for the public/ other health professionals who specialize in pain - Clearer communication- Within MDT (openness + understanding of allied health roles) - Case conferences held proactively to monitor patient progress. - Communication between all healthcare providers involved in the care of patients with chronic pain - GP networking with private pain specialists
4	Understanding and Awareness of Evidence Based Pain Management Approaches by Health Professionals	10%	- Training for all staff in ED to have a similar approach to the acute low back pain model so that medication is not always first line treatment or alone - Late referrals or no referrals from GP’s to other clinics- to manage complex chronic pain patients - Miscommunicating/ confused/conflicting advice from various health professional’s patient has seen. Strong focus on medical model rather than biosocialpsych model of pain - Use of non evidence based practice: “Low value care”: By other HPW’s as well as other PT’s
5	Access to Care	9%	- Waitlist time for access to services allows problems to get worse - Being able to deliver appropriate level of care to patients with low level of resources/ $/ travel - Difficulties knowing which practitioners are pain specialists. No database to find these specialists - Building more pain programs, pain clinics, easier access - Tele-health for rural services - Tele-health services
6	Physical therapy Advocacy and Leadership in Pain Management	9%	- Lobby government for change - Profession feels ignored/ uphill battle - Take ownership of MSK issues ie. See physio first instead of pain specialists > take leadership role in MDT - For allied health to take a stronger role and to reduce role of “biomedical” in pain services - To lobby for changes in Medicare/ PBS funding models - Champion research, distribution of research findings quicker
7	Understanding and Awareness of Evidence Based Pain Management Approaches in the General Community	6%	- Poor health literacy of public health patients - Community expectations/ awareness of chronic pain – of our patients is different to the level of results we will realistically achieve. - Release information brochure/ booklet for community to let them know that physios can help with pain - Reduce social stigma regarding chronic pain. Eg). Like recent efforts/ profile of mental health - Community engagement/ promotion of physios in Rx of pain. Eg). Professional bodies, famous people, ambassadors for change, discussion forums for consumers
8	Patient Engagement and Expectations	4%	- Challenge of meeting patient/ referral expectations - Expectations of patient of what treatment techniques they can get from physio. Eg). People want a massage even if its not evidence based - Expectations of other health professionals: physios will “fix” you. Expectations also from patient - Patient expectations: strong beliefs and reliance on certain modalities to cope with their pain - Compliance, engaging long-term
9	Clinical Time	4%	- Not enough time - Time: Initial time to take history; length of consultation/ episode duration/ service restrictions - Prioritizing/ allocating more time with chronic pain patients - Complexity of patient’s vs time constraints when seeing patients
10	Emotional Wellbeing of clinicians	4%	- Burnout/ emotional burden of seeing chronic patients - Exhausting work for physios- physically, emotionally- can lead to burnout - Frustration of not ‘curing’ a patient – moving from ‘cure’ model to a ‘management’ model - As a profession- Supervision (wellbeing) not embedded into practice - Mental exhaustion of physio when seeing chronic patients who may have psychological/ trauma/ PTSD issues large proportion of Rx is psychological (Pt depressed, sick role)> physios don’t receive much training in undergrad degree - Feelings of rejection - Expectations of needing to “fix” symptoms - Lack of clear career progression
11	Patient Clinical and Social Complexity	3%	- Complexity of presentation: younger clients, Trauma background, Indigenous, mental health problems - Patients are complex! - Mental exhaustion of physio when seeing chronic patients who may have psychological/ trauma/ PTSD issues large proportion of Rx is psychological (Pt depressed, sick role)> physios don’t receive much training in undergrad degree - Dealing with pts who have experienced pain for 10+ years - NESB patients with chronic pain- communicating with patients that don’t speak English

### Overarching theme - the complexity of pain

The complexity of persistent pain, including a poor understanding of the underlying causes and therefore the potentially unlimited targets for treatment, was clearly identified as a key factor that impacts the care of patients experiencing persistent pain across all system levels.

### Themes

#### Clinical skills and education

There was a perceived inadequacy of training and education about pain science and pain management approaches. Participants suggested that there is a lack of training in more psychologically informed techniques including motivational interviewing, counselling, and trauma skills. Participants reported that they perceived undergraduate physical therapy degrees to lack an appropriate amount of training specific to persistent pain and associated communication skills.

Solutions included increased education in training programs on persistent pain, more student placements in persistent tertiary pain clinics, and enhanced post graduate skills based professional development to provide opportunity for mastery and application of skills. A mixture of online and local opportunities was identified to support upskilling. Finally, shadowing and debriefing would enhance clinical practice through the sharing of successful clinical skills and techniques.

#### Funding models

Issues relating to funding models and systems were consistently documented. Concerns about the costs to patient(s) associated with accessing health care were raised, with reference to the inadequate allied health rebates and clinical guidelines that fund low value models of care such as heat and massage. Increased administrative load associated with compensable care was identified, with paperwork and challenging communication with insurers mentioned as issues impacting on physical therapists.

Solutions offered by the groups related to an increase in funding and resources to adequately support services for people experiencing persistent pain through dedicated pain programs, both in public, and private services. It was suggested by one group that an increase in staffing trained in a biopsychosocial approach to pain management is required.

#### Professional networking and referring of patients

This theme focused on the networking of physical therapists with other physical therapists and medical professionals to support better care of patients through more efficient triaging and diagnosis of patients. Responses emphasized that physical therapists should work within connected networks, collaborating, consulting, and sharing knowledge with other physical therapists and members of the medical team rather than practicing in isolation. This interconnected care extends into more effective communication amongst multidisciplinary teams which is achieved through better networking. Effective databases of appropriate physical therapists and other medical professionals would assist in effective networking.

#### Understanding and awareness of evidence based pain management approaches by health professionals

A lack of understanding and awareness of evidence-based pain management approaches among health professionals is a challenge. Inconsistent and poor advice, low value care, and non-evidence-based practice were highlighted.

Suggestions for opportunities to overcome these barriers included improved training and education of general practitioners and other health professionals in regards to persistent pain both at undergraduate and post graduate levels.

#### Access to care

The access to appropriate, timely care is a key issue especially for public services, which may lead to a worsening of the pain condition/presentation. Inadequate availability of health professionals in community settings particularly in rural areas was noted as a challenge.

Several suggestions were made to improve patient access to care. The use of telehealth services and training for physical therapists to be able to harness telehealth effectively was supported to increase accessibility. Improving referral pathways and availability of specialist pain services, such as through telehealth, may help to alleviate access issues. Additionally, enhancing multidisciplinary care in the primary care setting through increased connections between health professionals was suggested. The creation of a database of physical therapists and other health professionals with knowledge and training in pain management may improve awareness and access to appropriate services.

#### Physical therapy advocacy and leadership in pain management

Participants indicated that to improve advocacy and leadership, physical therapists should advocate both for their patients, and for broader changes within the health system. Furthermore, there should be increased promotion of the role of physical therapists in pain management, and physical therapists should take more of a leadership role in multidisciplinary teams. Many opportunities highlighted by the groups related to the role that physical therapists may play in leadership and advocacy both within and outside of the physical therapy profession. Physical therapists should take a leadership role, particularly for musculoskeletal conditions, with reference made to *Physio First* models of care i.e., physical therapist as ‘First Contact Practitioners’ in primary care.^23^

#### Understanding and awareness of evidence based pain management approaches in the general community

Barriers relating to the understanding of pain and awareness of pain management approaches were identified not just amongst health professionals, but also in the broader community. It was highlighted that people may have preconceived beliefs about what pain is and what pain management should involve, expecting passive modalities over active management. It was noted that these beliefs and expectations often conflict with evidence-based approaches to pain management, and may be challenging to navigate, particularly in private settings.

The need for a public-health approach, with “whole population education” and strategies to facilitate “behavior change” at a broader level was suggested as well as the use of digital mediums (including “social media”, “internet”, and “YouTube”) to improve awareness of evidence-based pain management approaches.

#### Patient engagement & expectations

Participants identified that people experiencing persistent pain have specific expectations of physical therapy that often did not align with current management approaches. These expectations can also extend to other health professionals. Patients may have an expectation they will be fixed by the physical therapist or that specific modalities will be used, which can result in disengagement from care.

#### Clinical time

Limited clinical time available to clinicians was identified as an issue. Participants indicated that patients with persistent pain require more time than other patients in order to address the complexity of patient needs and to allow for effective case formulation and management.

#### Emotional wellbeing of clinicians

The complexity of patients can contribute to an emotional burden on the clinician. The incidence of trauma and psychological issues experienced by people experiencing persistent pain was highlighted as a contributing factor to the emotional wellbeing of clinicians. Inadequate clinical support such as debriefing not being embedded in practice were also factors.

#### Patient clinical and social complexity

People experiencing persistent pain were identified as complex, who often had a long duration of symptoms alongside complex psychological and social histories. It was suggested that factors including the presence of trauma, mental health disorders, and cultural and linguistic diversity contribute to challenges of managing patients experiencing persistent pain. Challenging behaviors associated with opioid access was identified as another potential aspect of the complex social environment that physical therapists need to navigate in order to provide effective support and management.

## Discussion

The aim of this study was to explore the experiences of Australian Physical therapists when working with people who experience persistent pain. The complexity of pain was the overarching theme that permeated throughout the data. We identified eleven interrelated themes that were not mutually exclusive, demonstrating considerable overlap. The eleven overlapping themes reflect pain as a complex condition within a similarly complex multi‑level health and social system.

Although we found that the themes overlapped, there were three key themes that physical therapists reported as critical to managing people experiencing persistent pain. While all eleven themes were interrelated, these three were consistently described across the majority of participant groups and appeared with higher coverage in the written data (Table 3). Participants frequently linked system-level barriers related to funding with limitations in clinical education and skills, reinforcing these themes as central leverage points for improving care. For this reason, we focused the discussion on these priority areas while acknowledging that meaningful change requires action across the entire system. These were the complexity of pain, clinical skills and education, and funding models. These findings are supported by a systematic review by Ng and colleagues^24^ who found parallel findings of the barriers (e.g., clinician skill and knowledge, funding models, and organizational factors) to health professionals adopting a biopsychosocial approach to musculoskeletal pain and how the barriers are interwoven through the clinical, service, and system levels.

### The complexity of pain

Persistent pain has long been considered a complex human condition with no clear pathoanatomical cause and recognition for an approach that considers a non-biomedical perspective.^25^, ^26^, ^27^, ^28^ Physical therapists clearly identified that pain is a complex experience, that is managed in a complex system. While not a novel finding, we felt it was important to highlight and anchor the rest of the results under this overarching theme raised by the participants.

Over recent decades the shift towards the biopsychosocial model features heavily in the literature as a prevailing model in which to consider persistent pain.^29^, ^30^, ^31^, ^32^ The model allows physical therapists an approach to make sense of complexity when patients present with persistent pain.^33^, ^34^, ^35^ Our study highlights that it is not just the complexity of the patients and their pain, but also their interactions within the complexity of the broader system they operate in that leads to significant challenges.

### Clinical skills and education

Our results indicated that the evidence shift to more psychologically informed physical therapy interventions has not been supported with appropriate training for physical therapists. It was recognized that training courses alone are not sufficient for the clinical application of such interventions and that ongoing mentoring and shadowing of other physical therapists are needed. These results are supported by evidence that recommends a structured training approach to psychologically informed physical therapy underpinned by strong communication skills.^36^, ^37^, ^38^ Indeed, there is emphasis on the importance of training in applying psychologically informed physical therapy,^39^ that does come with its own challenges, particularly where psychology supervision is not readily accessible.^40^^,^^41^ These studies underscore the need for psychologically informed physical therapy training to effectively integrate theoretical skills into practice.

Indeed, there was a perception that pre-registration training programs do not adequately prepare graduate physical therapists to support the management of people experiencing persistent pain, highlighting the need for earlier education in pain science and management. The role of pain science has been described as a potential threshold concept in physical therapy training programs and there is a curriculum available from the IASP that can be successfully implemented in such programs.^42^^,^^43^ Given the complex and multidimensional nature of pain, it is understandable that training programs have faced challenges in incorporating theoretical aspects, such as perceptual, ontological, epistemological, linguistic, and existential dimensions, into physical therapy education. In addition, with the scope of practice continuing to expand and the demands of the curriculum already high, it is difficult to expose students to and train them in evidence-based interventions within the constraints of current programs.^43^ Practice thresholds need to be achievable and balance the ability to appropriately recognize, triage, and provide initial evidenced based care to people experiencing persistent pain while aligning with post graduate training opportunities and specialist pathways.

### Funding models

Our results indicate that there is a lack of funding and resources that impacts on the delivery of appropriate care for people experiencing persistent pain, and that changes in funding structures need to occur to support the increased consultation times required. The issues identified mirror those in a study conducted in Israel arguing that the time allocated combined with the increased administrative burden was a barrier to effective management.^44^ While evidence broadly supports multidisciplinary, biopsychosocial interventions for persistent pain, funding models to support the delivery of such interventions to a broad number of people is lacking.^1^^,^^45^

### A systems approach

A systems approach enables the identification of solutions by addressing overlapping issues and finding opportunities for convergence, rather than using reductionist methods that focus on isolated solutions for each issue. Because the themes reflected a complex system rather than discrete categories, some conceptual overlap is unavoidable and reflects how participants themselves described interconnected challenges.

There has been a call for greater recognition and application of complexity theory in health care, and rethinking reductionist, linear approaches to addressing health issues in favor of adopting a complex adaptive systems perspective.^46^ This approach conceptualizes health outcomes as the emergent product of a complex system, comprised of smaller nested systems of interconnected “agents” at various levels of scale (ranging from single cells and body systems, to organisms, to communities, institutions, and government), where the individual is viewed not in isolation but embedded within a broader context, environment, and society.^47^ Previous literature has proposed the application of systems perspectives to the phenomena of pain,^48^^,^^49^ which may offer an opportunity to better appreciate the complexity of factors contributing to individual experiences of pain, as well as the global burden pain represents and the role of physical therapists as being part of the solution.

**Fig. 1:**
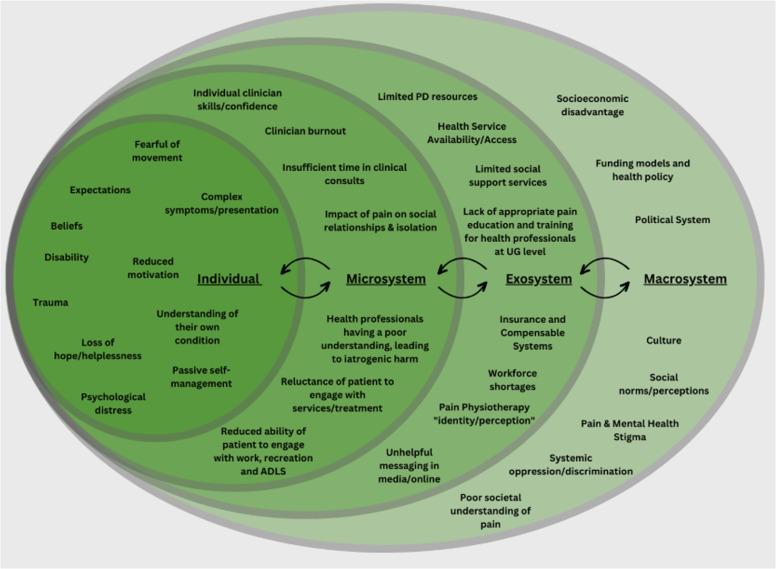
Challenges and barriers identified by Australian Physical therapists when working with patients with pain.

Nested systems can be used to understand factors influencing outcomes at various levels, ranging from the individual to society as a whole. Drawing on and adapting Bronfrenbrenner’s Ecological Systems theory,^50^ we plot the themes of this study at the levels of the individual (i.e., the patient), the “microsystem” (i.e., the patient's immediate environment, the places they go and the people they interact with, including health professionals and services), “exosystem” (i.e., organizations, local governments, broader community, health and educational systems, infrastructure, media/internet), and “macrosystem” (i.e., policy, culture, social norms and perceptions, systemic injustice and oppression) levels (Fig. 1). We have chosen to omit the “mesosystem” level as described by Brofrenbrenner, as it encapsulates the interactions between components of the microsystem and is arguably redundant given that systems theory implies inherent interrelationships between agents within and between all levels of a system.

When we consider the key findings from our study we see that the themes extend across all levels of the system. Funding, for example, impacts the individual through their financial means, the microsystem through the payment options and funding provided to the health care provider, the exosystem through the insurance and compensation schemes, and the macrosystem, through cultural expectations and health care policy effected through state and federal budgets. Change and interventions are required throughout all levels of the system and cannot be viewed only in isolation.

Likewise, while clinical skills and education are the responsibility of the individual, they are influenced by the workplace and its emphasis on providing employee education. University training within the exosystem must also align with professional accreditation requirements, with funding made available to support this training.

When considering solutions to these challenges, enablers that extend across systems should be considered. For example, the Australian Physical therapy Association is an entity that has the ability to impact the individual through the training they offer to patients (e.g., seminars, website) and clinicians (e.g., professional development, grants), but can also lobby governments for targeted funding for health and telehealth schemes to increase access for patients and increased funding for extended consultation times for people experiencing persistent pain. For instance, the Australian government's National Strategic Action Plan for pain management has allocated early funding to train health professionals and develop an education strategy to improve access to consistent, up-to-date information and programs for managing persistent pain.^51^

While individual clinicians may typically orient towards individual and microsystem-level solutions (e.g., within the direct therapeutic context or through professional development and networking), there have been recent calls for physical therapists to adopt a “transformational view,” and consider how society might be changed to address broader social determinants of health. McGrath and colleagues^52^ state, “ignoring systematic problems reinforces the status quo…physical therapists should engage in transformational change by attempting to alter the system they perceive to be unjust.” Changes and interventions that span all levels of the system, from individual to macro-systems are required to ensure interventions are effective and appropriately address the complexity of pain and the challenges that exist in managing it.

### Limitations

It is important to recognize the limitations of this study. First, the data was collected prior to the COVID-19 pandemic and given the significant impact of COVID-19 on the health system and society, it is likely that these perspectives may not fully reflect the current experiences of Australian physical therapists. The pandemic shifted care delivery, increasing telehealth options, many which have become permanent, addressing the backlog of surgical rehabilitation, and having to integrate long COVID care into their practice, all exacerbating access and availability.^53^

Next, while not strictly a limitation, the inherent bias of the pragmatic approach should be acknowledged. This cohort represents an almost exclusively metropolitan subset of physical therapists and given that data collection occurred at the physical therapy specific Pain Day 2019, it could be assumed that most if not all have a high degree of interest in the area of persistent pain. In addition, coverage data should be interpreted with caution, as it reflects the proportion of written text by the scribe and not necessarily the entirety of the conversations across the groups. We acknowledge that this approach may limit narrative depth; however, the use of structured templates, multiple scribes, and comparative review helped mitigate the risk of scribe bias and loss of meaning. Our results should not be considered representative of the entire physical therapy profession. Future research in this area should include experiences of physical therapists who reside in rural and remote areas that experience inequitable health burden, and those who work across a variety of settings and clinical interest areas. Given this is a study focusing on the experiences of physical therapists, it is not surprising that many of the suggestions to improve practice involved advocacy, leadership, and promotion of the role of physical therapists in pain management.

Last is the potential bias stemming from the authors' positionality as physical therapists who specialize in pain management and hold academic roles in the field. Their deep involvement in the profession and subject matter may have influenced the development of the research questions, interpretation of the data, and analysis. While their expertise provides valuable insight, it may also predispose them to certain assumptions about pain management that could shape the data collection process, potentially affecting the objectivity of their interpretation. This positionality may also influence how they interpret the participants' responses, given the shared professional background and language, possibly leading to a biased confirmation of pre-existing beliefs or frameworks about pain management in physical therapy. It is important to acknowledge that their shared professional experiences with the participants could shape the dynamics of the group discussions, as participants may have felt encouraged to align their perspectives with the predominant professional discourse.

### Conclusion

Ultimately, the findings of this study reinforce the sentiments of broader research that systems-informed approaches to tackling the issue of persistent pain are vital. The insights provided by physical therapists in this study offer nuanced and contextualized perspectives on the realities of working directly with people experiencing persistent pain in the Australian context, and afforded the opportunity for generative, bottom-up suggestions for how barriers and challenges might be addressed at various system levels. Future research should embrace a complex systems perspective to best address the problem of persistent pain and seek novel opportunities to improve outcomes. As Kuhn highlights, “complexity fosters reflection and thoughtfulness…. complexity in this way does not offer research recipes, ‘tried and true’, but rather a space for thinking otherwise”.

## Declaration of competing interest

The authors declare no competing interest.

## Appendix A

### Instructions for the groups

You will NOT be audio recorded or video recorded during the small group discussion.

### Scribe instructions

As the scribe it is your responsibility to document the discussion and main topics for each question.

You do not need to write down everything that is said, but instead document the key topics that are addressed.

Try to remain as unbiased as possible.

For each question, after 8 min you will be asked to read back to the group the main points you have documented and check the notes accurately represent the thoughts and discussion of the group.

For each question ensure each person has been given an opportunity to provide input.

### Participant instructions

1. The primary investigator will ask for volunteers for someone to act as a scribe in each group.
2. In your group spend 10 min discussing each of the four questions below.
3. You will be given a prompt after 5 and 8 min have elapsed for each question. After 10 min you will be asked to move onto the next question.
4. You will have 10 min after the fourth question to amend or change any information the scribe has noted down
5. When discussing the questions avoid focusing on issues that are specific to one site due to individual procedural or personal concerns. Instead focus on issues that are more general.
6. Each person will be given 30 s at the start of each question to share their immediate thoughts.
7. After 8 min the scribe will read back the key points that were documented and check what has been recorded accurately reflects the thoughts of the group.

Each group will independently discuss the questions and provide a summary of the discussion and key points from their group.

You will have 10 min to discuss each question.

One person from each group will self-nominate as a scribe and summarize the key points for each topic on a supplied form.

## Appendix B

### Participant information details

Please answer all the questions below only after you have read, understood, signed and completed the participant information sheet and consent form:

1. Participant Gender:
  Female
  Male
  Other: Please specify- _______________________________________
2. How would you describe your place of employment where you encounter patients with chronic pain? (Please tick only one)
  Public pain clinic- Primary
  Public pain clinic- Secondary
  Private pain clinic
  General private practice clinic
  Public hospital (not specialist pain unit)
  Other (Please specify)
  _______________________________________________________________ _
3. What is the postcode of the place of employment indicated above?
  _______________________________________________________________ _
4. For how many years have you been practicing as a physical therapist?
  _______________________________________________________________ _
5. In thinking about your daily practice what percentage of patients are treated under a third party or workers compensation scheme?
  _______________________________________________________________ _
